# Health Related Quality of Life and Sleep Regularity Among Middle‐Aged to Older Adults From the Community

**DOI:** 10.1111/jsr.70194

**Published:** 2025-09-03

**Authors:** Kelly Sansom, Murthy M. N. Mittinty, Hannah Scott, Bastien Lechat, Daniel Windred, Andrew J. K. Phillips, Robert Adams, Peter R. Eastwood, Amy C. Reynolds

**Affiliations:** ^1^ Centre for Healthy Ageing, Health Futures Institute Murdoch University Perth Western Australia Australia; ^2^ Flinders Health and Medical Research Institute (Sleep Health), College of Medicine and Public Health Flinders University Adelaide South Australia Australia

**Keywords:** irregular sleep, mental health, physical health, sleep consistency, sleep patterns

## Abstract

Irregular sleep is increasingly related to poorer health, with stronger links to cardiovascular disease and mortality than sleep duration. Its impact on health‐related quality of life, however, remains unclear, particularly in community‐based populations. This study examined whether objectively measured sleep regularity is associated with physical and mental health‐related quality of life. Sleep regularity was calculated using the Sleep Regularity Index from actigraphy data in 768 middle‐aged to older adults from the Raine Study (median age [range] = 57 [53–61]; 58% female). Physical and mental health‐related quality of life were assessed using the 12‐item Short Form Health Survey. Quantile regression was used to examine associations at the 25th, 50th, and 75th percentiles, adjusting for age, sex, comorbidity count, sleep duration, and shift work. Median sleep regularity scores declined with self‐rated health, from 77.17 (excellent) to 61.49 (poor). A 10‐unit increase in sleep regularity was associated with higher mental health scores at the 25th (1.80; 95% CI: 0.90–2.60), 50th (1.20; 95% CI: 0.50–1.90), and 75th (0.50; 95% CI: 0.20–0.90) percentiles. For physical health, a 10‐unit increase in sleep regularity was associated with a 1.20 (95% CI: 0.30–2.20) higher score at the 25th percentile, with no evidence of association at higher percentiles. These findings suggest that poorer sleep regularity is related to lower physical and mental health‐related quality of life. Future research should explore whether improving sleep regularity can enhance quality of life in middle‐aged to older adults.

## 1 Background

Occupational and social commitments can disrupt the maintenance of regular sleep patterns (i.e., sleep regularity) (Taillard et al. 2021). Not adhering to a regular sleep pattern can lead to misalignment between sleep patterns and the endogenous circadian rhythm, causing sleep difficulties and daytime impairments. Such misalignment of circadian rhythms is commonly observed in shift workers and those who routinely have different sleep and wake times during the week versus weekends. Studies have shown that both shift workers and individuals with more variation in their sleep and wake times have poorer mental health and health‐related quality of life (HRQOL) (Islam et al. 2019; Chang and Jang 2019; Kim et al. 2002; Torquati et al. 2019). However, the impact of sleep regularity on HRQOL in community populations is less understood (Sletten et al. 2023).

The Sleep Regularity Index (SRI) captures day‐to‐day changes in sleep–wake patterns and is associated with adverse health outcomes including increased mortality and physical health issues (Sletten et al. 2023; Windred et al. 2024; Fischer et al. 2021; Lunsford‐Avery et al. 2018). The SRI is also a stronger predictor of health outcomes than other dimensions of sleep health such as sleep duration (Windred et al. 2024). Despite its potential relevance, research on the relationship between SRI and HRQOL remains limited to one prior study in cancer patients. This study found that lower SRI scores were linked to poorer HRQOL, suggesting that sleep regularity may influence subjective well‐being even in populations already burdened by illness (Trivedi et al. 2021). This is particularly important in community populations, where sleep irregularity may be overlooked but still contribute meaningfully to reduced well‐being. HRQOL is increasingly recognised as a key outcome in clinical care including guiding treatment decisions at both individual and population levels (Phyo et al. 2020). Investigating its relationship with sleep regularity in general populations may help identify novel targets for improving well‐being and inform future research and public health strategies.

The current study aimed to describe and investigate the associations between the SRI and HRQOL in middle‐aged adults from the Raine Study. We firstly aimed to describe the distribution of the SRI and its relationship with self‐reported overall health rating. Secondly, the association between SRI and HRQOL across different levels of HRQOL distribution was examined using quantile regression (Staffa et al. 2019).

## 2 Methods

### 2.1 Participants

**FIGURE 1:**
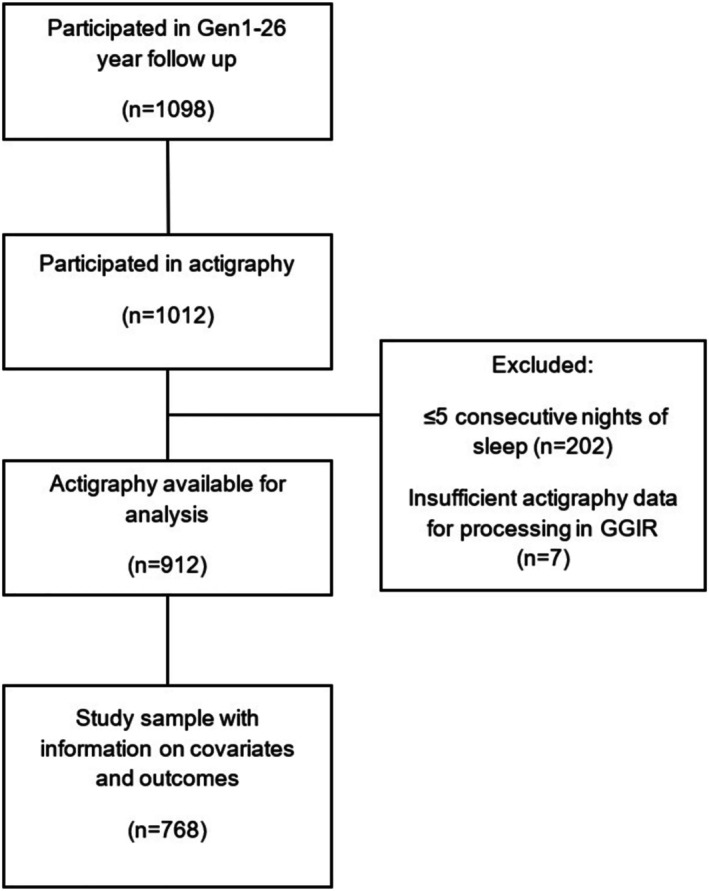
Represents the Raine Study middle‐aged community sample who had complete information relevant to the present study.

This study included Generation 1 (Gen1) participants from the Raine Study, who are the biological parents of the original birth cohort (Generation 2, Gen2). Although only Gen1 participants were assessed, follow‐up waves in the Raine Study are named according to the age of Gen2 participants. The data used in this study were collected during the Gen1‐26‐year follow‐up, which corresponds to the time when Gen2 participants were 26 years old. Detailed descriptions of Gen1 participants are available elsewhere (Dontje et al. 2019). This follow‐up involved comprehensive sleep assessments including in‐laboratory polysomnography and 1 week of actigraphy monitoring in the home setting. Additionally, participants completed health and lifestyle questionnaires, including information on HRQOL. The Raine Study data collection was approved by the University of Western Australia, RA/4/1/5202, and the present analysis was approved by Flinders University Human Research Ethics Committee (HEL4717‐2). A flow diagram of participants included in the current study amply is summarised in Figure 1.

### 2.2 Measures

#### 2.2.1 Actigraphy

Wrist actigraphy (GT3X + ActiGraph LLC, Pensacola, FL; sampling frequency 30 Hz; idle sleep mode not enabled) was collected in participants for a one‐week period during everyday life. The actigraphy data were processed in R package *GGIR* (version 2.00) to derive sleep and wake information. The processing and cleaning of the GGIR files are detailed in a previously published manuscript (Sansom et al. 2023). Sleep and wake times were derived from the GGIR HDCZA algorithm (van Hees et al. 2015).

#### 2.2.2 Sleep Regularity Index

Sleep regularity was measured using the SRI, which quantifies how regular sleep and wake timing is from 1 day to the next. This is calculated as the probability of a person being awake compared to being asleep at two timepoints, 24 h apart. Scores range from 0 (completely random sleep–wake patterns) to 100 (perfectly regular timing). In a previous population study of middle to older aged adults, the median score was 81 with an interquartile range of 74–86 (Phillips et al. 2017).

The SRI scores were derived from actigraphy‐based sleep and wake data in the home setting for up to 7 days. Sleep and wake times were estimated in the open‐source R packages ‘GGIR’ (version 2.0.0) and ‘sleepreg’ (version 1.3.5). The ‘sleepreg’ package uses the sustained inactivity bouts determined from GGIR to determine naps, fragmented sleep, and long periods of awaking that were not considered in GGIR alone (Windred et al. 2021). Sleepreg also considers device non‐wear and excludes sleep and wake times miscalculated by GGIR (Windred et al. 2021). We included participants in analyses who had 5 or more 24‐hperiods of valid information as indicated by the *sleepreg* output (Fischer et al. 2021).

### 2.3 Health‐Related Quality of Life

The HRQOL was assessed by the short‐form 12 (SF‐12, version 2.0). The SF‐12 is a 12‐item questionnaire that assesses the impact of eight domains of health (physical functioning, physical role limitation, pain, general health, vitality, social functioning, mental health and role limitation emotional) on everyday life (Ware Jr. et al. 1996). Mental and physical composite score summary scales were computed from the SF‐12 questionnaire, termed mental HRQOL and physical HRQOL. The SF‐12 has demonstrated strong psychometric properties, including internal consistency with Cronbach's alpha > 0.80 for both physical and mental component scores, and validity through high correlations with SF‐36 scores and other health measures in its original validation study (Ware Jr. et al. 1996).

The questionnaire was scored using freely accessible code provided by the University of California (Hays Pages—Ron Hays: Programs/Utilities (ucla.edu)). The mental and physical composite score summary utilises a norm‐based scoring system to interpret the mental composite score and physical composite scores, with a mean of 50 and a standard deviation of 10 in the general population. This means that a score above 50 indicates a better‐than‐average HRQOL, while scores below 50 suggest below‐average health. As a part of the SF‐12, participants were asked to rank their general health as either excellent, very good, good, fair, or poor.

### 2.4 Health and Lifestyle Information

Information on a wide range of health comorbidities and sleep health assessments was collected in the Gen1‐26‐year follow‐up. Specific conditions of interest in this study were selected to compare HRQOL among different severities of sleep regularity to HRQOL in major health conditions including coronary heart disease, anxiety and depressive symptoms, and chronic obstructive pulmonary disease. Coronary heart disease was defined by self‐reported doctor‐diagnosed coronary heart disease or stroke, including angina, pacemaker implants, myocardial infarction, carotid surgery, coronary angioplasty or stent, and coronary bypass. Anxiety and depressive symptoms were assessed by the General Anxiety Disorder‐7 (GAD‐7) (Phillips et al. 2017) and Patient Health Questionnaire (PHQ‐9) (Windred et al. 2021) with moderate to severe symptoms defined as a score ≥ 10, respectively. Chronic obstructive pulmonary disease was defined as responding ‘Yes’ to a doctor diagnosis of the condition.

Other health and lifestyle evaluations were included for description and to address confounding. Confounders were defined a priori using directed acyclic diagrams (DAGs), presented in Figure S1. Health comorbidities were assessed based on a ‘Yes’, ‘No’, or ‘Unsure’ response to doctor‐diagnosed conditions, including angina, claudication, high blood pressure, high cholesterol, myocardial infarction, transient ischemic attack, stroke, diabetes, obstructive sleep apnea, narcolepsy, insomnia, restless legs or periodic leg movements, depression, anxiety, chronic obstructive pulmonary disease, and cancer in the past 5 years. Responses of ‘Unsure’ were coded as ‘No’ based on the assumption that major health events likely to impact quality of life would be memorable to participants. A count of total comorbidities was computed, categorising participants as having 0, 1, or ≥ 2 comorbidities. Sex was reported as biological sex at birth. Shift work status was determined by whether participants responded ‘Yes’ or ‘No’ to ‘Are you a shift worker?’. Mean habitual sleep duration from the main sleep episode in a 24‐h period was derived from actigraphy data processed and analysed in GGIR, as described in the actigraphy methods. Moderate to severe obstructive sleep apnea was defined as an apnea hypopnea index of ≥ 15 events/h based on in‐laboratory polysomnography (American Academy of Medicine 2012 scoring criteria) (Berry et al. 2012) and insomnia symptoms (Insomnia Symptom Questionnaire) (Okun et al. 2009). Further description of sleep disorders in the Gen1's has previously been published (McArdle et al. 2022).

### 2.5 Statistical Analyses

The relationship between SRI scores and self‐reported general health and HRQOL was described using boxplots and bar charts, respectively. We firstly compared the median and interquartile range (IQR) of SRI and self‐reported health groups (excellent, very good, good, fair and poor). In Supporting Information, the SRI score was categorised into four bins (0–59, 60–69, 70–79, > 80) and the median HRQOL scores across the SRI groups were compared. While 10‐point intervals were preferred, the number of participants with scores < 60 and > 80 was small. As no established thresholds exist, these categories were chosen pragmatically to balance sample size and descriptive clarity. Additionally, mean HRQOL scores in people with doctor‐diagnosed coronary heart disease, depression symptoms, anxiety symptoms, and chronic obstructive pulmonary disease were included as comparators to aid clinical interpretation. The effect size difference between each of the groups was calculated using Cohen's D with an effect size of < 0.5 considered small, 0.5–< 0.8 as medium, and ≥ 0.8as large (Lakens 2013).

The relationship between SRI and the 25th, 50th, and 75th percentiles of HRQOL scores was examined using quantile regression. Quantile regression was also used to examine associations between SRI and HRQOL across the HRQOL outcome distribution. All models were adjusted for covariates to reduce confounding bias as identified a priori using directed acyclic diagrams (Figure S1). We included a sensitivity analysis without including the count of comorbidities as this could potentially be an effect of SRI, further details reported in the supplement. We reported coefficients and 95% confidence intervals for each quantile regression model. All results were presented based on a 10‐unit change in the SRI score, as a single unit change is considered too small to be meaningful. Statistical analyses were performed in RStudio (RStudio Team 2018, Boston, MA) using R version 4.0.4 (R Core Team 2021, Vienna, Austria).

## 3 Results

### 3.1 Participant Characteristics

Participants (*n* = 768) had a median age of 56.90 (IQR, 53.10–60.60) years, 58% were female, and 48% reported no significant health comorbidity. Eleven percent of the participants engaged in shift work and participants slept a median of 6.85 (IQR, 6.27–7.36) hours per night during a one‐week period of home actigraphy monitoring. The median number of days of recordings per person was 7.02 (IQR, 6.05, 7.08) (Table 1).

**TABLE 1:** The Raine study generation1–26‐year follow‐up participants included in the study sample.

Characteristic	*N* = 768^a^
Age	56.9 (53.1, 60.6)
Female	443 (58%)
Count of comorbidities	
0	364 (47%)
1	242 (32%)
≥ 2	162 (21%)
Shift work	86 (11%)
Sleep duration (actigraphy)	6.86 (6.28, 7.36)
Ethnicity	
Caucasian (European descent)	707 (92%)
Aboriginal	3 (0.4%)
Polynesian	2 (0.3%)
Vietnamese	1 (0.1%)
Chinese	23 (3.0%)
Indian	29 (3.8%)
Other	2 (0.3%)
Unknown	1
Number of days of actigraphy recordings to calculate SRI, days	7.02 (6.05, 7.08)
Insomnia symptoms	117 (15%)
Moderate to severe obstructive sleep apnea	232 (30%)
Coronary heart disease	34 (4.4%)
Cancer diagnosis in the last 5 years	56 (7.3%)
Chronic obstructive pulmonary disease	7 (0.9%)
Severe anxiety symptoms	57 (7.4%)
Severe depressive symptoms	53 (6.9%)

^a^ Median (IQR); *n* (%).

### 3.2 SRI Distribution

**FIGURE 2:**
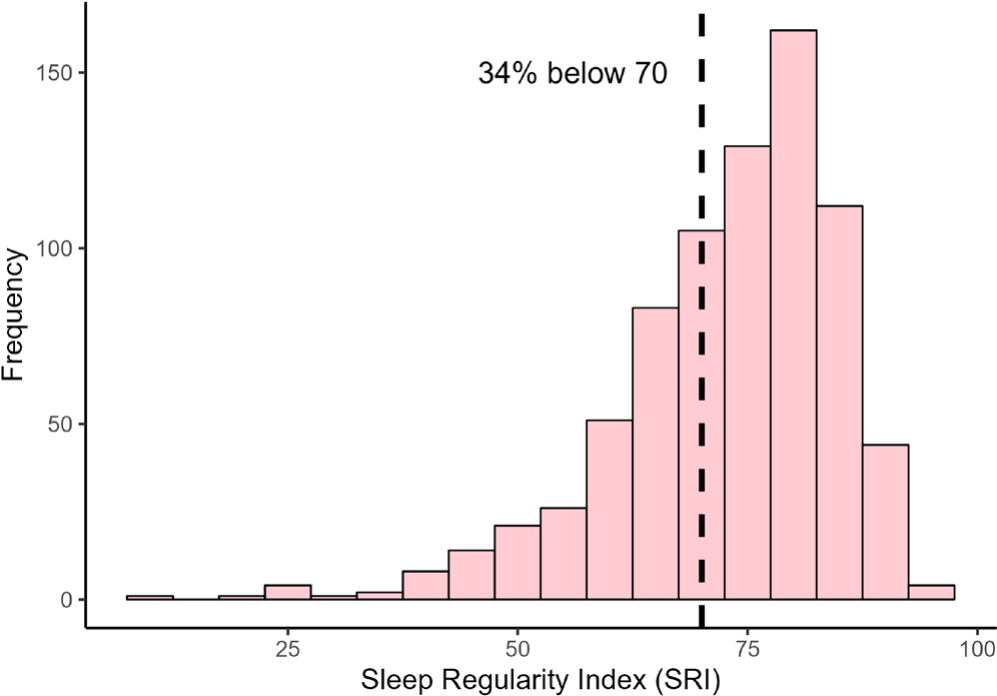
Distribution of Sleep Regularity Index (SRI) scores in the study sample of the Raine Study middle‐aged community cohort.

**FIGURE 3:**
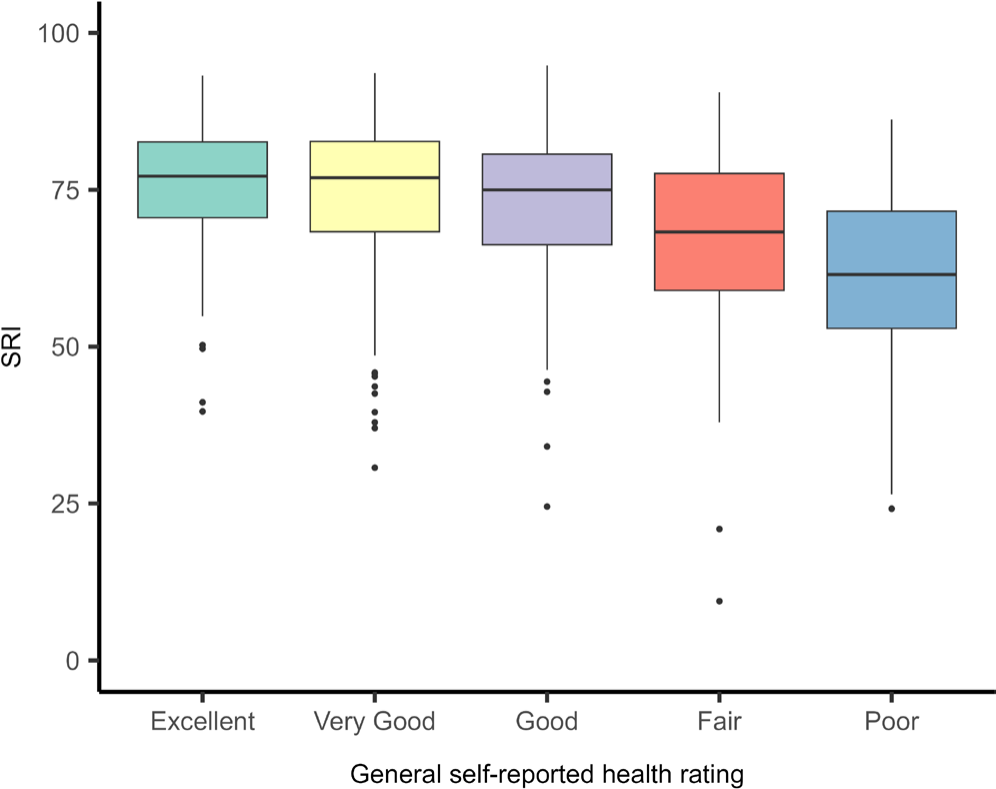
Boxplot of Sleep Regularity Index (SRI) scores by general self‐reported health rating.

The median SRI score was 72.75 (IQR, 66.30, 81.50) and ranged from 9.44 to 94.80. The distribution was skewed to the left, with more individuals reporting higher regularity scores. The prevalence of individuals with an SRI score ≤ 70 was 34% (Figure 2). A summary of SRI scores by self‐reported health category is presented in Figure 3. The median SRI scores were 77.17 (IQR, 70.57, 82.63), 76.92 (IQR, 68.32, 82.71) and 74.97 (IQR, 66.24, 80.67) among those reporting excellent, very good, and good health. Those reporting fair and poor health had lower SRI scores, poorer sleep regularity, being 68.28 (IQR, 58.97, 77.60) and 61.49 (IQR, 52.93, 71.58), respectively.

### 3.3 Health Comorbidities Across Sleep Regularity Severities

**FIGURE 4:**
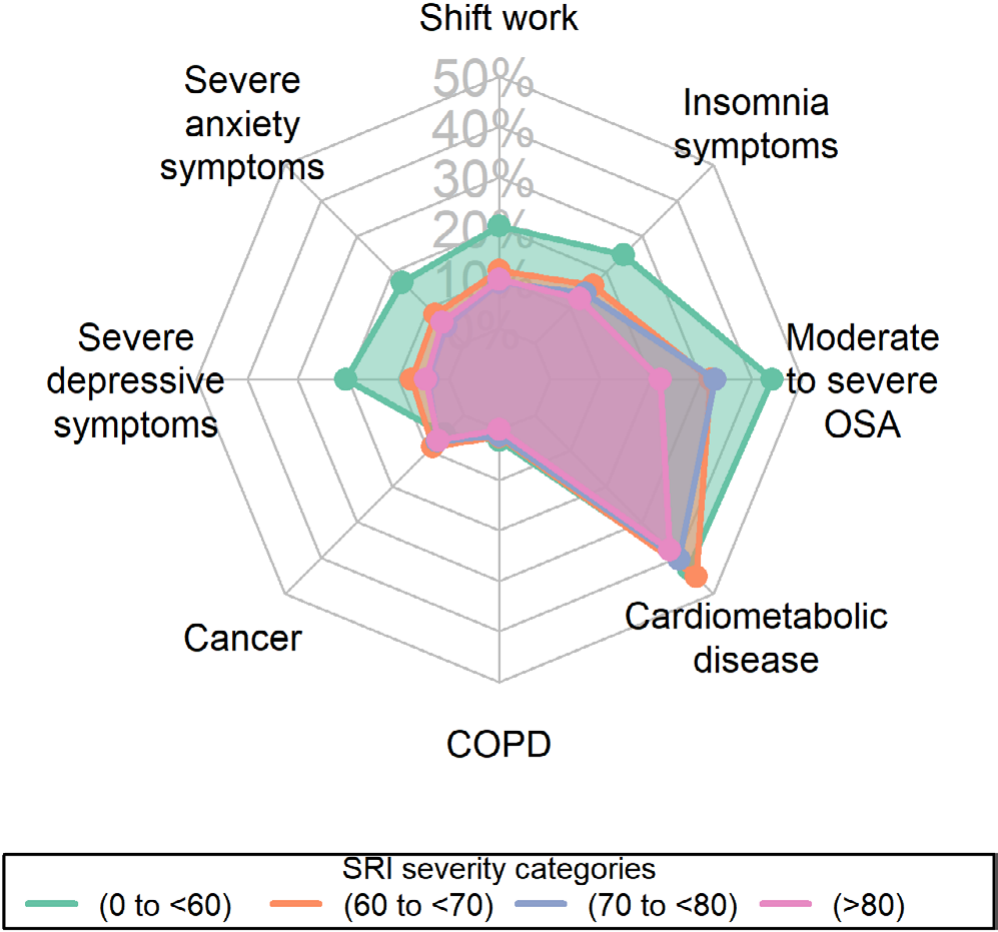
Radar chart of comorbidity prevalence by Sleep Regularity Index (SRI) severity categories.

The prevalence of common health conditions among sleep regularity severity categories is summarised in Figure 4 and Table S1. Individuals with the lowest sleep regularity scores (range, 0–59) had the highest prevalence of insomnia symptoms (25%), obstructive sleep apnoea (44%), depressive symptoms (20%) and anxiety symptoms (17%) while individuals with regularity scores above 80 had the lowest prevalence (12%, 22%, 4.5% and 6%, respectively, further details provided in Table S1). The mean HRQOL scores across different SRI severity groups and other common health conditions are plotted in Figure S2. Individuals in the lowest SRI severity group had HRQOL scores lower than the standardised mean of 50, while among participants with SRI scores greater than 70 had above average (> 50 HRQOL score) mental and physical HRQOL.

### 3.4 HRQOL Across Sleep Regularity Groups and Other Major Conditions

Mean differences in mental and physical HRQOL across sleep regularity categories and major health conditions (coronary heart disease, chronic obstructive pulmonary disease, depression symptoms and anxiety symptoms) are presented in Tables S3 and S4. Those with the lowest SRI scores (0–59) had mean mental and physical HRQOL below the standardised mean of 50, while higher SRI score categories were above the mean. The effect size for mental HRQOL was moderate between the lowest SRI scores (0–59) and scores ≥ 70, while for physical HRQOL, it was moderate between SRI scores (0–59) and ≥ 80. Physical HRQOL differences were large between all SRI groups and COPD. Lower SRI scores (< 70) had a small effect size difference in physical HRQOL from CHD and depression symptoms, whereas SRI scores > 70 had moderately higher physical HRQOL scores relative to CHD and depression symptoms.

### 3.5 Mental and Physical HRQOL

**FIGURE 5:**
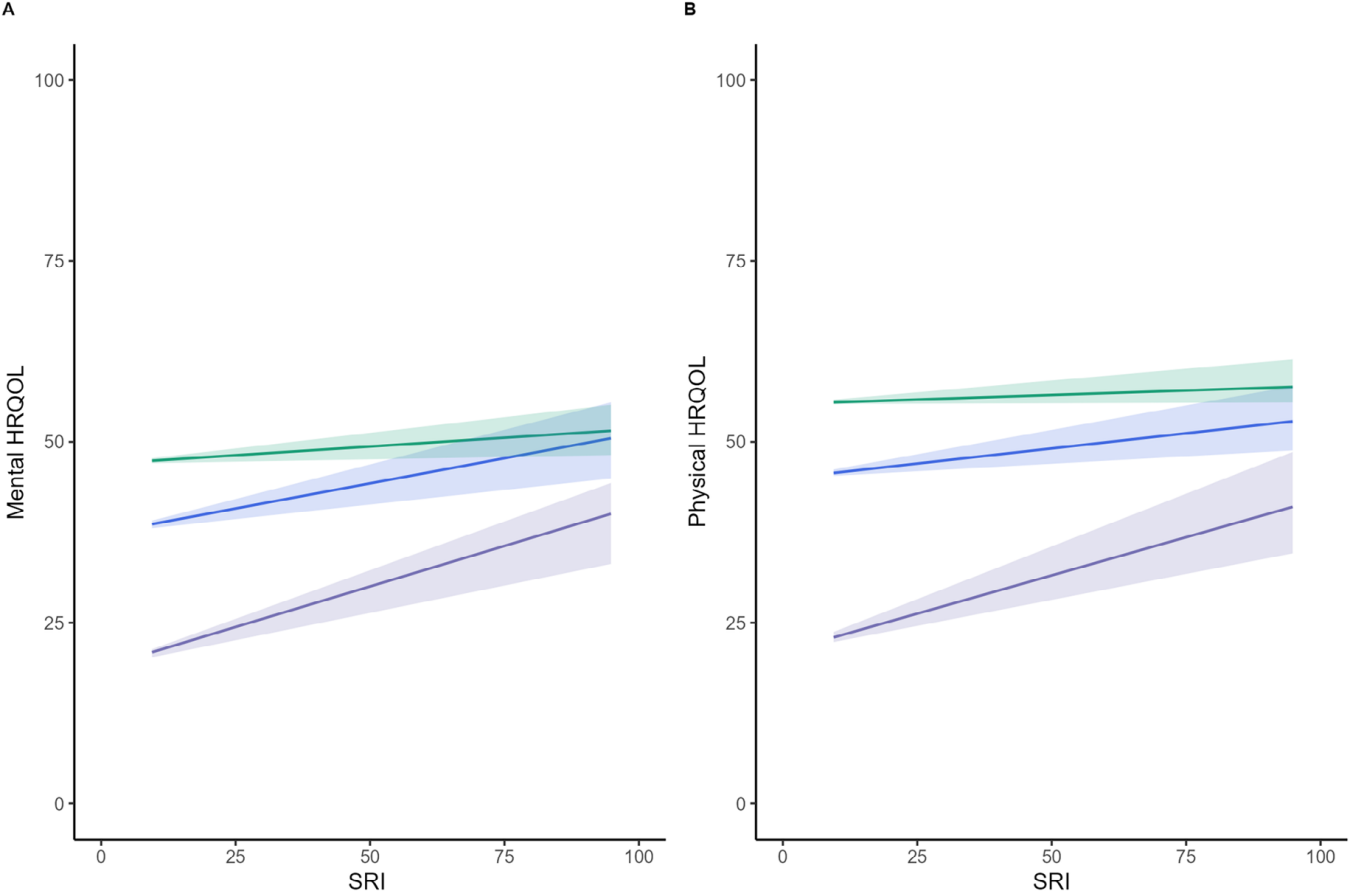
Quantile regression of health‐related quality of life (HRQOL) by the sleep regularity index (SRI). The outcomes depicted in each panel are: (A) mental health HRQOL and (B) physical health HRQOL. All models were adjusted for sex, habitual sleep duration, shift work, and number of comorbidities. The red, green and blue lines represent the lower 25th, middle 50th and upper 25th percentiles of HRQOL.

The quantile regression analysis of SRI on the mental and physical components of HRQOL revealed differential associations across quantiles (Figure 5). For mental HRQOL, a 10.00‐unit increase in SRI was associated with a 1.80‐unit increase at the 25th percentile (coefficient: 1.80, 95% CI: 0.90–2.60), a 1.20‐unit increase at the 50th percentile (coefficient: 1.20, 95% CI: 0.50–1.90), and a 0.50‐unit increase at the 75th percentile (coefficient: 0.50, 95% CI: 0.20–0.90). For physical HRQOL, a 10.00‐unit increase in SRI was associated with a 1.20‐unit higher score at the 25th percentile (coefficient: 1.20, 95% CI: 0.30–2.20). However, there was no strong evidence of an association of SRI on physical HRQOL at the 50th (coefficient: 0.20, 95% CI: −0.4 to 0.70) and 75th (coefficient: 0.30, 95% CI: 0.00–0.80) percentiles of the HRQOL distribution. The sensitivity analysis of the association between SRI and HRQOL without count of comorbidities as a confounder is presented in Table S2.

## 4 Discussion

In this large community sample of middle‐aged to older adults, individuals with lower sleep regularity scores reported having poorer overall general health status. Shift work, insomnia symptoms, depression, anxiety, and moderate to severe sleep apnea were more prevalent among those with lower relative to higher sleep regularity scores. Importantly, a relationship was found between the SRI and HRQOL, with higher regularity associated with better HRQOL, particularly among those reporting poorer HRQOL.

### 4.1 Distribution of SRI and HRQOL

The distribution of SRI scores in this study is comparable to distributions reported in other large cohort studies of middle‐aged to older adults, such as the United Kingdom Biobank (UKB) (Windred et al. 2021) and the United States‐based Multi‐Ethnic Study of Atherosclerosis (Lunsford‐Avery et al. 2018) (MESA). Thirty‐four percent of the SRI scores were below 70. This is a significant proportion of people potentially at risk of poor health outcomes as findings from the UKB have shown SRI of < 70 is associated with higher risk of all‐cause mortality (Windred et al. 2024) and major adverse cardiovascular events (Chaput et al. 2025). Furthermore, the distribution of SRI scores varied with self‐reported health status. Participants reporting fair to poor health had median SRI scores of 69 and 62, respectively, which are notably lower than the median SRI score of 77 among those reporting excellent health.

In sensitivity analysis, the mean HRQOL scores varied across SRI score categories and major health conditions. Individuals with SRI scores < 60 had a standardised mental and physical HRQOL below the standardised mean, indicating poorer mean HRQOL relative to those with SRI scores > 60. The effect size difference was moderate when comparing mean HRQOL in those with SRI < 60 to those scoring > 70. For CHD and depression, SRI scores < 70 had similar physical HRQOL, while scores > 70 showed moderately higher HRQOL. This indicates the mean HRQOL of people with SRI scores < 70 was comparable to people with CHD and depression symptoms. Individuals with COPD, which is often associated with significantly poorer HRQOL (Huber et al. 2015), as expected, had the largest effect difference in mean physical HRQOL relative to all SRI categories.

### 4.2 SRI and Mental and Physical HRQOL

Lower SRI scores were associated with reduced perceived mental and physical HRQOL. This relationship varied across different quantiles of HRQOL such that individuals with lower HRQOL showed a stronger positive association between sleep regularity and HRQOL, whereas this relationship, although still significant, was weaker among those with higher HRQOL scores. These findings align with previous research in cancer patients, where lower sleep regularity was linked to poorer cancer‐related QoL (Trivedi et al. 2021). While specific mental health conditions were not measured in the present study, other research has shown that irregular sleep patterns are associated with poor mental health and functioning, including: PTSD (Mascaro et al. 2021); mental health in adolescents (Castiglione‐Fontanellaz et al. 2023); daytime functioning in delayed sleep–wake phase disorder (Murray et al. 2019); relapse in alcohol use disorder (Barb et al. 2022); substance use and prospective depression (Walsh et al. 2025); and self‐injury (Burke et al. 2022). Similarly, previous research has shown lower sleep regularity was associated with physical health outcomes, including cardiometabolic disease (Zhang and Qin 2023; Chaput et al. 2024) sleep apnea (Sansom et al. 2024), and mortality (Windred et al. 2024). Unlike prior investigations, this study focused on patient‐reported health. Assessing patient‐reported health outcomes is vital, as it ensures healthcare and research focus on factors that directly impact how patients experience and evaluate their own health.

### 4.3 Potential Mechanistic Pathways

Sleep irregularity may contribute to poorer health‐related quality of life (HRQOL) through its potential to disrupt circadian rhythms. Irregular sleep patterns have been associated with delayed circadian timing and altered light exposure rhythms, suggesting potential disruption of the circadian system (Phillips et al. 2017). The circadian system, together with the homeostatic sleep drive, regulates alertness, emotional regulation, and cardiometabolic and immune function (Fishbein et al. 2021). Disruption of these systems may lead to hormone dysregulation and altered neurotransmitter activity, potentially contributing to symptoms of depression and anxiety (Walker 2nd et al. 2020). A recent study found irregular sleep to be associated with mistimed light exposure, which previously irregular light exposure is a driver of circadian disruption (Hand et al. 2023) and mistimed light exposure has been associated with adverse mental health, metabolic dysfunction, and cardiovascular outcomes (Burns et al. 2023; Bonatto et al. 2024). While these mechanisms were not directly assessed in our study, they offer a plausible framework for interpreting the observed associations and identifying directions for future research. Longitudinal studies are needed to clarify causal pathways and to account for other behavioural factors such as meal timing and physical activity that may influence sleep regularity.

### 4.4 Strengths and Limitations

Strengths of this study include: the involvement of participants from the community who represent Australian middle‐aged to older adults, thereby making findings generalisable to the broader Australian community; controlling for shiftwork in our modelling, which is important as shiftwork contributes to disrupted sleep patterns; the use of the SRI, which captures day‐to‐day changes in sleep patterns and may therefore be a more sensitive marker of circadian disruption than other measures such as standard deviation in sleep times; and the exploration of associations between HRQOL outcomes as opposed to mean values, thereby allowing examination of associations across the full range of the HRQOL distribution.

While this study provides valuable insights, it is important to acknowledge methodological limitations. One potential limitation of the study is the assessment of regularity over a one‐week period, which does not permit comment on any long‐term relationships between sleep patterns and HRQOL, which might be particularly important given sleep regularity appears to vary by season across the year (Scott et al. 2025). Additionally, we were unable to calculate Cronbach's alpha for the SF‐12, which limits our ability to comment on the psychometric properties of this instrument within our sample. Another limitation is the participants being mainly Caucasian; thus, the results cannot be generalised to ethnically diverse populations.

## 5 Conclusion

In this cohort of middle‐aged to older adults, we found a strong association between sleep regularity and HRQOL, with poorer sleep regularity linked to lower perceived mental and physical health. This relationship was particularly pronounced among those with poorer HRQOL, underscoring the potential importance of sleep regularity in contributing to health disparities. The potential for a larger impact on HRQOL at the lower end of the spectrum suggests non‐linear effects, where individuals with poorer HRQOL might experience more significant benefits from improved sleep regularity.

## Author Contributions

**Kelly Sansom:** conceptualization, methodology, software, formal analysis, visualization, project administration, writing – original draft, writing – review and editing. **Murthy M. N. Mittinty:** formal analysis, software, writing – review and editing, methodology, supervision. **Hannah Scott:** conceptualization, writing – review and editing, methodology. **Bastien Lechat:** conceptualization, methodology, writing – review and editing. **Daniel Windred:** methodology, writing – review and editing. **Andrew J. K. Phillips:** methodology, writing – review and editing. **Robert Adams:** methodology, supervision, writing – review and editing. **Peter R. Eastwood:** methodology, supervision, writing – review and editing. **Amy C. Reynolds:** methodology, conceptualization, writing – review and editing, formal analysis, supervision, project administration.

## Ethics Statement

The Raine Study data collection was approved by the University of Western Australia, RA/4/1/5202, and the present analysis was approved by Flinders University Human Research Ethics Committee (HEL4717‐2).

## Conflicts of Interest

The authors declare no conflicts of interest.

## Supporting information


**Data S1:** Supporting information.

## Data Availability

We are willing to share data from this study, according to current Raine study data sharing rules. The Raine Study holds a rich and detailed collection of data gathered over 30 years for the purpose of health and well‐being research. The informed consent provided by each participant does not permit individual‐level data to be made available in the public domain (i.e., a public data repository). However, de‐identified analytic data sets are available to all researchers for original research or auditing of published findings. All data access is managed through established Raine Study procedures, which require data handlers to agree to a code of conduct, outlined in the Raine Study Researcher Engagement Policy, that includes safeguards to protect the identity of participants. Details of the data access processes and code of conduct are available on the Raine Study website (www.rainestudy.org.au).
