# Supplementary material for: Health Related Quality of Life and Sleep Regularity Among Middle‐Aged to Older Adults From the Community
Source: J Sleep Res. 2025 Sep 3;35(2):e70194. doi: 10.1111/jsr.70194 (PMC13003304; doi:10.1111/jsr.70194)
Supplement: Supplementary file 1 — Data S1: Supporting information. [file JSR-35-e70194-s001.docx]

# Supplementary material

**Title:** Health related quality of life and sleep regularity among middle-aged adults in a community population

**Authors:** Kelly Sansom^1,2^, M.N. Mittinty^2^, Hannah Scott^2^, Bastien Lechat^2^, Daniel Windred^2^, Andrew J. K. Phillips^2^, Robert Adams^2^, Peter R Eastwood^1^ and Amy Reynolds^2^

1. Centre for Healthy Ageing, Health Futures Institute, Murdoch University, Perth, Western Australia
2. Flinders Health and Medical Research Institute (Sleep Health), College of Medicine and Public Health, Flinders University, Adelaide, South Australia

Corresponding author: Kelly Sansom

Email: Kelly.sansom@flinders.edu.au

Address: Level 2A Mark Oliphant Building, 5 Laffer drive Bedford Park, 5042, Adelaide, South Australia

| **Table S1: Prevalence of comorbidities by sleep regularity index (SRI) severity categories** | | | | |
| --- | --- | --- | --- | --- |
|  | **SRI severity categories** | | | |
| **Characteristic** | **(0 to <60)**, N = 93*^1^* | **(60 to <70)**, N = 148*^1^* | **(70 to <80)**, N = 262*^1^* | **(>80)**, N = 265*^1^* |
| Shift work | 19 (20%) | 17 (11%) | 24 (9.2%) | 26 (9.8%) |
| Severe anxiety symptoms | 16 (17%) | 12 (8.1%) | 13 (5.0%) | 16 (6.0%) |
| Severe depressive symptoms | 19 (20%) | 11 (7.4%) | 11 (4.2%) | 12 (4.5%) |
| Cancer diagnosis in the last five years | 5 (5.4%) | 13 (8.8%) | 19 (7.3%) | 19 (7.2%) |
| Coronary obstructive pulmonary disease | 2 (2.2%) | 2 (1.4%) | 3 (1.1%) | 0 (0%) |
| Coronary heart disease | 40 (43%) | 67 (45%) | 106 (40%) | 100 (38%) |
| Moderate to severe obstructive sleep apnea | 41 (44%) | 47 (32%) | 86 (33%) | 58 (22%) |
| Insomnia symptoms | 23 (25%) | 24 (16%) | 37 (14%) | 33 (12%) |
| *^1^* Median (IQR); n (%) | | | | |

| **Table S2: Linear regression of sleep regularity index on percentiles of mental and physical HRQOL** | | | | | | | | | |
| --- | --- | --- | --- | --- | --- | --- | --- | --- | --- |
| **Outcome** | **25^th^ percentile** | | | **50^th^ percentile** | | | **75^th^ percentile** | | |
|  | **Beta** | **95% CI***^1^* | **p-value** | **Beta** | **95% CI***^1^* | **p-value** | **Beta** | **95% CI***^1^* | **p-value** |
| Mental HRQOL | 0.22 | 0.11, 0.34 | <0.001 | 0.14 | 0.07, 0.21 | <0.001 | 0.05 | 0.01, 0.09 | 0.013 |
| Physical HRQOL | 0.21 | 0.11, 0.32 | <0.001 | 0.08 | 0.03, 0.14 | 0.004 | 0.03 | -0.01, 0.06 | 0.2 |
| *^1^* CI = Confidence Interval  Adjusted for age, sex, sleep duration and shift work | | | | | | | | | |

**Confounding**

The main analysis of the association between SRI and HRQOL was adjusted based on confounding paths identified in Figure S1A. To reduce confounding conditioned on sleep duration, sex, age, count of comorbidities and shift work. Count of comorbidities was considered a confounder on the basis that people with more health problems will have more disrupted sleep leading to irregular schedules and such conditions would also increase HRQOL. It is also possible that sleep regularity is a causal factor in developing more comorbidities in which case comorbidities could be a mediator on the pathway to HRQOL. However, we do not have sufficient causal evidence to confirm this therefore we performed the main analysis by adjusting on count of comorbidities and a sensitivity analysis without adjusting for comorbidities, on variables as shown in Figure S1B.

Figure S1: Directed acyclic diagram

A)


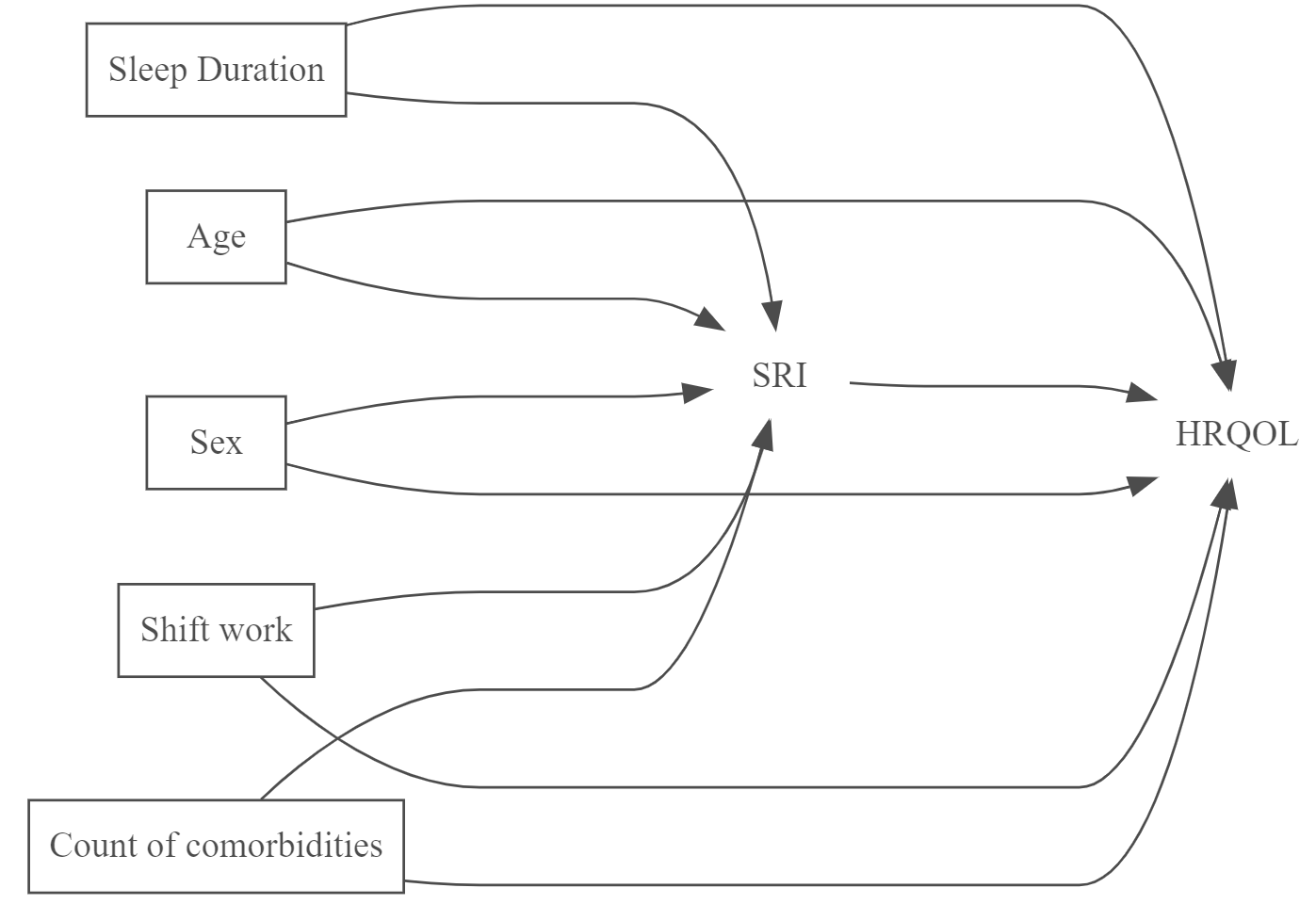


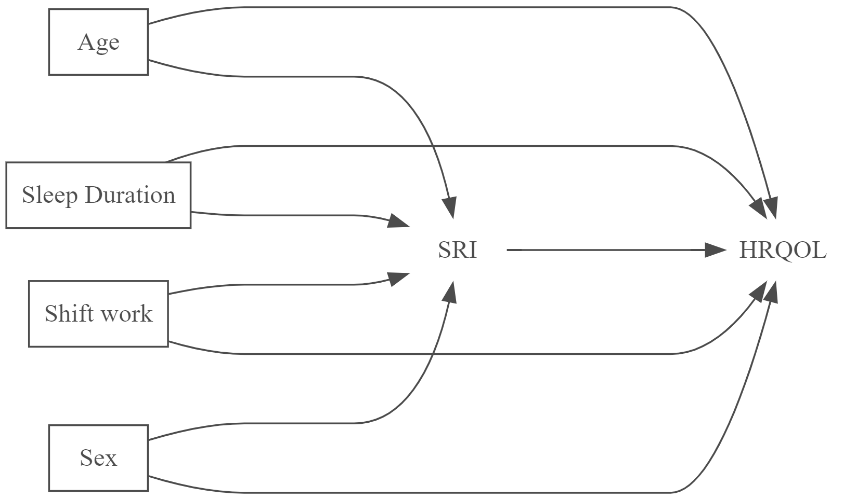
B)


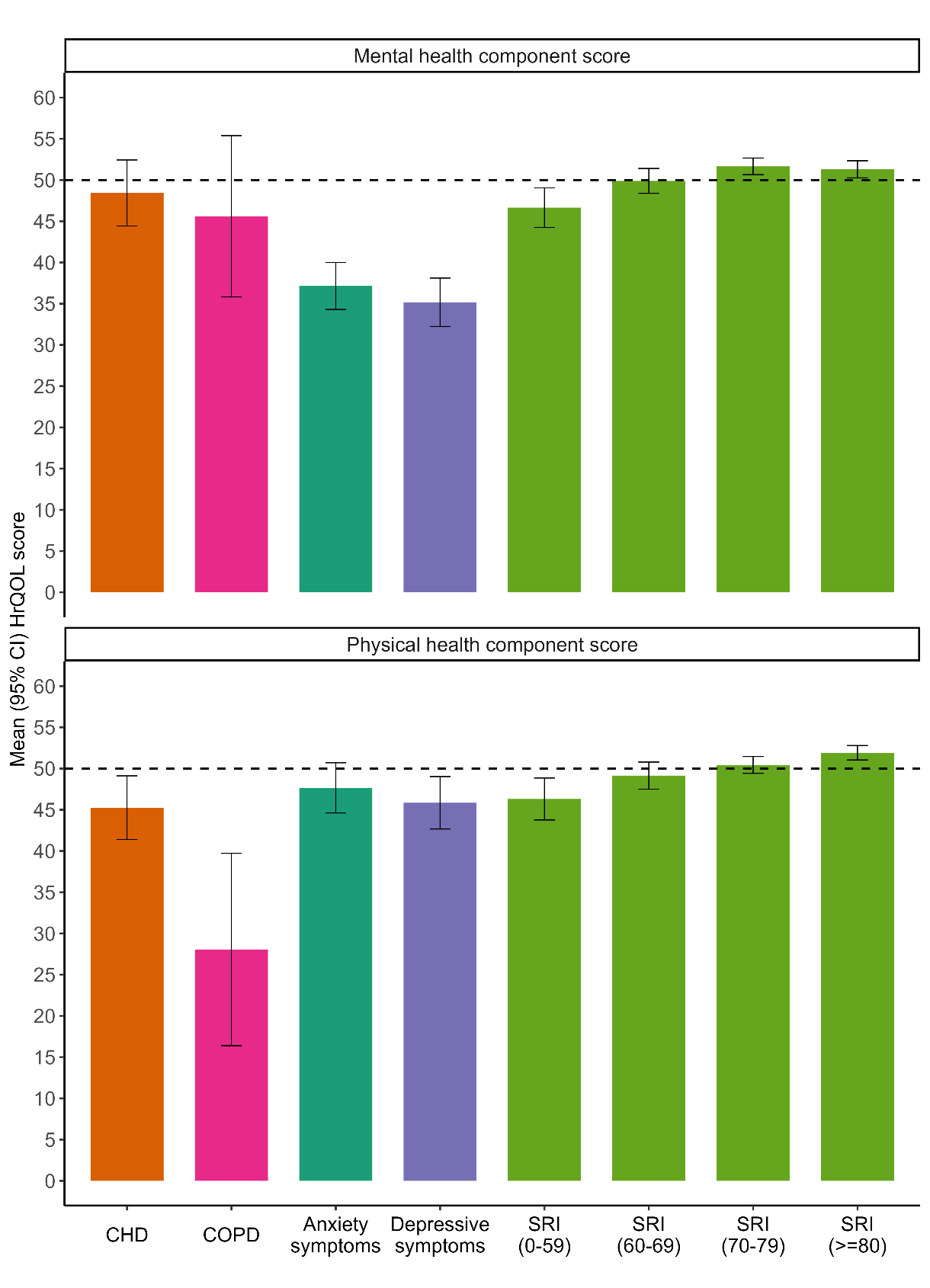
Figure S2: **Comparison of mean (95% CI) health related quality of life (HRQOL) scores across sleep regularity index (SRI) severities and other common health conditions**. The dashed line represents the standardized mean score of 50, where values above indicate better-than-average health and values below indicate below-average health.CI= confidence interval, CHD = coronary heart disease, COPD = chronic obstructive pulmonary disease.

| **Table S3:** Comparison of mental health HRQOL scores among SRI groups and other major health conditions | | | | | |
| --- | --- | --- | --- | --- | --- |
| **SRI groups** | **Mean HRQOL (SD)** | **Comparator group** | **Mean HRQOL (SD)** | **Cohens D** | **Effect size** |
| SRI (0-59) | 46.65 (11.82) | CHD | 48.43 (11.91) | 0.2 | Small |
| SRI (0-59) | 46.65 (11.82) | COPD | 45.61 (13.21) | 0.1 | Small |
| SRI (0-59) | 46.65 (11.82) | Anxiety symptoms | 37.16 (10.99) | 0.8 | Large |
| SRI (0-59) | 46.65 (11.82) | Depressive symptoms | 35.17 (10.91) | 1.0 | Large |
| SRI (0-59) | 46.65 (11.82) | SRI (60-69) | 49.89 (9.35) | 0.3 | Small |
| SRI (0-59) | 46.65 (11.82) | SRI (70-79) | 51.66 (8.32) | 0.5 | Medium |
| SRI (0-59) | 46.65 (11.82) | SRI (>=80) | 51.3 (8.6) | 0.5 | Medium |
| SRI (60-69) | 49.89 (9.35) | CHD | 48.43 (11.91) | 0.1 | Small |
| SRI (60-69) | 49.89 (9.35) | COPD | 45.61 (13.21) | 0.4 | Small |
| SRI (60-69) | 49.89 (9.35) | Anxiety symptoms | 37.16 (10.99) | 1.3 | Large |
| SRI (60-69) | 49.89 (9.35) | Depressive symptoms | 35.17 (10.91) | 1.5 | Large |
| SRI (60-69) | 49.89 (9.35) | SRI (0-59) | 46.65 (11.82) | 0.3 | Small |
| SRI (60-69) | 49.89 (9.35) | SRI (70-79) | 51.66 (8.32) | 0.2 | Small |
| SRI (60-69) | 49.89 (9.35) | SRI (>=80) | 51.3 (8.6) | 0.2 | Small |
| SRI (70-79) | 51.66 (8.32) | CHD | 48.43 (11.91) | 0.3 | Small |
| SRI (70-79) | 51.66 (8.32) | COPD | 45.61 (13.21) | 0.6 | Medium |
| SRI (70-79) | 51.66 (8.32) | Anxiety symptoms | 37.16 (10.99) | 1.5 | Large |
| SRI (70-79) | 51.66 (8.32) | Depressive symptoms | 35.17 (10.91) | 1.7 | Large |
| SRI (70-79) | 51.66 (8.32) | SRI (0-59) | 46.65 (11.82) | 0.5 | Medium |
| SRI (70-79) | 51.66 (8.32) | SRI (60-69) | 49.89 (9.35) | 0.2 | Small |
| SRI (70-79) | 51.66 (8.32) | SRI (>=80) | 51.3 (8.6) | 0.04 | Small |
| SRI (>=80) | 51.3 (8.6) | CHD | 48.43 (11.91) | 0.3 | Small |
| SRI (>=80) | 51.3 (8.6) | COPD | 45.61 (13.21) | 0.5 | Medium |
| SRI (>=80) | 51.3 (8.6) | Anxiety symptoms | 37.16 (10.99) | 1.4 | Large |
| SRI (>=80) | 51.3 (8.6) | Depressive symptoms | 35.17 (10.91) | 1.6 | Large |
| Abbreviations: HRQOL = Health-related quality of life, SRI = sleep regularity index, CHD = coronary heart disease, COPD = coronary obstructive pulmonary disease, SD = standard deviation. | | | | | |

| **Table S4:** Comparison of physical health HRQOL scores among SRI groups and other major health conditions | | | | | |
| --- | --- | --- | --- | --- | --- |
| **SRI groups** | **Mean HRQOL (SD)** | **Comparator group** | **Mean HRQOL (SD)** | **Cohens D** | **Effect size** |
| SRI (0-59) | 46.31 (12.5) | CHD | 45.26 (11.49) | 0.1 | Small |
| SRI (0-59) | 46.31 (12.5) | COPD | 28.05 (15.75) | 1.3 | Large |
| SRI (0-59) | 46.31 (12.5) | Anxiety symptoms | 47.66 (11.71) | 0.1 | Small |
| SRI (0-59) | 46.31 (12.5) | Depressive symptoms | 45.85 (11.81) | 0.04 | Small |
| SRI (0-59) | 46.31 (12.5) | SRI (60-69) | 49.15 (10.16) | 0.3 | Small |
| SRI (0-59) | 46.31 (12.5) | SRI (70-79) | 50.44 (8.4) | 0.4 | Small |
| SRI (0-59) | 46.31 (12.5) | SRI (>=80) | 51.92 (7.27) | 0.6 | Medium |
| SRI (60-69) | 49.15 (10.16) | CHD | 45.26 (11.49) | 0.4 | Small |
| SRI (60-69) | 49.15 (10.16) | COPD | 28.05 (15.75) | 1.6 | Large |
| SRI (60-69) | 49.15 (10.16) | Anxiety symptoms | 47.66 (11.71) | 0.1 | Small |
| SRI (60-69) | 49.15 (10.16) | Depressive symptoms | 45.85 (11.81) | 0.3 | Small |
| SRI (60-69) | 49.15 (10.16) | SRI (0-59) | 46.31 (12.5) | 0.3 | Small |
| SRI (60-69) | 49.15 (10.16) | SRI (70-79) | 50.44 (8.4) | 0.1 | Small |
| SRI (60-69) | 49.15 (10.16) | SRI (>=80) | 51.92 (7.27) | 0.3 | Small |
| SRI (70-79) | 50.44 (8.4) | CHD | 45.26 (11.49) | 0.5 | Medium |
| SRI (70-79) | 50.44 (8.4) | COPD | 28.05 (15.75) | 1.8 | Large |
| SRI (70-79) | 50.44 (8.4) | Anxiety symptoms | 47.66 (11.71) | 0.3 | Small |
| SRI (70-79) | 50.44 (8.4) | Depressive symptoms | 45.85 (11.81) | 0.5 | Medium |
| SRI (70-79) | 50.44 (8.4) | SRI (0-59) | 46.31 (12.5) | 0.4 | Small |
| SRI (70-79) | 50.44 (8.4) | SRI (60-69) | 49.15 (10.16) | 0.1 | Small |
| SRI (70-79) | 50.44 (8.4) | SRI (>=80) | 51.92 (7.27) | 0.2 | Small |
| SRI (>=80) | 51.92 (7.27) | CHD | 45.26 (11.49) | 0.7 | Medium |
| SRI (>=80) | 51.92 (7.27) | COPD | 28.05 (15.75) | 2.0 | Large |
| SRI (>=80) | 51.92 (7.27) | Anxiety symptoms | 47.66 (11.71) | 0.4 | Small |
| SRI (>=80) | 51.92 (7.27) | Depressive symptoms | 45.85 (11.81) | 0.6 | Medium |
| Abbreviations: HRQOL = Health-related quality of life, SRI = sleep regularity index, CHD = coronary heart disease, COPD = coronary obstructive pulmonary disease, SD = standard deviation. | | | | | |
